# Genetic Factors and the Risk of Periodontitis Development: Findings from a Systematic Review Composed of 13 Studies of Meta-Analysis with 71,531 Participants

**DOI:** 10.1155/2017/1914073

**Published:** 2017-04-26

**Authors:** Maélson Klever da Silva, Antonio Carlos Gonçalves de Carvalho, Even Herlany Pereira Alves, Felipe Rodolfo Pereira da Silva, Larissa dos Santos Pessoa, Daniel Fernando Pereira Vasconcelos

**Affiliations:** ^1^Laboratory of Analysis and Histological Processing (LAPHIS), Federal University of Piaui, Parnaiba, PI, Brazil; ^2^Postgraduate Program in Biomedical Sciences, Federal University of Piaui, Parnaiba, PI, Brazil; ^3^Postgraduate Program in Biotechnology, Federal University of Piaui, Parnaiba, PI, Brazil; ^4^Postgraduate Program in Dentistry, Federal University of Piaui, Teresina, PI, Brazil

## Abstract

*Purpose*. This work aimed to synthesize the results of recent meta-analysis focusing on polymorphism in inflammatory mediators and its relation with the risk of periodontitis development.* Materials and Methods*. A systematic search was conducted using databases for publications prior to October 2016. Three examiners extracted data from articles with a clear association between polymorphisms in the inflammatory mediator gene and the development of periodontitis through meta-analysis using the fixed or randomized statistical models to calculate the Odds Ratio with values of *P* < 0.05 considered significant.* Results*. A total of 13 meta-analysis articles with 25 polymorphisms in seven interleukins (IL-1A, IL-1B, IL-4, IL-6, IL-8, IL-10, and IL-18), three cellular receptors (Fc*γ* receptors: FCGR2A, FCGR3A, and FCGR3B), and five inflammatory mediators (COX-2, MMP-2, MMP-3, MMP-8, and MMP-9), with a total of 71,531 participants, approaching different classifications of the disease.* Conclusion*. The study demonstrated that polymorphisms in the IL-1A, IL-1B, IL-6, IL-10, MMP-3 (chronic form), and MMP-9 (chronic form) polymorphisms were significantly associated with the risk of developing periodontitis, whereas other polymorphisms in the IL-4, IL-8, IL-18, Fc*γ*, COX-2, MMP-2, MMP-3 (aggressive), MMP-8, and MMP-9 (aggressive) polymorphisms had no significant association with risk of developing periodontitis.

## 1. Introduction

Periodontitis is a multifactorial inflammatory disease and both environmental and genetic factors play a major role in the progression of the disease with consequent tissue destruction around the dental roots, and alveolar bone is associated with systemic alterations such as diabetes [1], changes in the liver [2], cardiovascular diseases [3], and even osteoporosis [4].

The high risk in the progression of periodontitis is directly associated with the biofilm found in the gingival sulcus, in which both amount and presence of specific species of bacteria represent risk factors [5]. However, the genetic variability of host may influence individual susceptibility to disease development, so as to determine the clinical aspects and rate of periodontitis progression.

The evidence that periodontitis is a complex disease of multifactorial etiology has resulted in the development of focused researches in the identification of molecular markers capable of determining the risk of disease development [6].

Recently, investigations on factors of susceptibility to periodontitis have been gaining focus on genes of immunoregulatory molecules, such as cytokines, chemokines, membrane surface receptors, and antigen recognition proteins [5]. Cytokines such as interleukins (IL-1A, IL-1B, IL-6, and IL-10, among others), surface receptors such as the Fc*γ* family (FCGRs), and cyclooxygenase- (COX-) 2 and matrix metalloproteinase (MMP) are considered key factors in the progression of periodontitis recruitment, differentiation and activation of B lymphocytes, inflammatory infiltrate, and stimulation of osteoclasts. Polymorphisms in these molecules have been suggested as factors that influence the risk of developing the disease [7–11], although several studies using allelic and genotype frequency determination methods by Polymerase Chain Reaction (PCR) [12] or genomic association [13] have sought to clarify the relationship between polymorphisms in cytokines or other inflammatory mediators and periodontitis, if failures by the reduced sample number generate false-positive or false-negative data [5].

In this perspective, meta-analysis studies have been increasingly used in genetic analysis for their ability to associate data by canceling the so-called short effects that studies with reduced sample numbers lead to [14]. Meta-analyses on polymorphisms in inflammatory mediators are available in the literature [15–17]. The high capacity of association of data made by the meta-analysis guarantees greater reliability as well as accurate results with correct orientation of the focus in the development of molecular markers for the disease.

Given such reliability in meta-analysis studies, this study aimed to describe the association between polymorphisms in inflammatory mediators and the susceptibility to periodontitis based on available meta-analysis in the literature.

## 2. Methods

This meta-analysis followed the recommended PRISMA (Preferred Reporting Items for Systematic Reviews and Meta-Analyses) statement [26].

**Figure 1:**
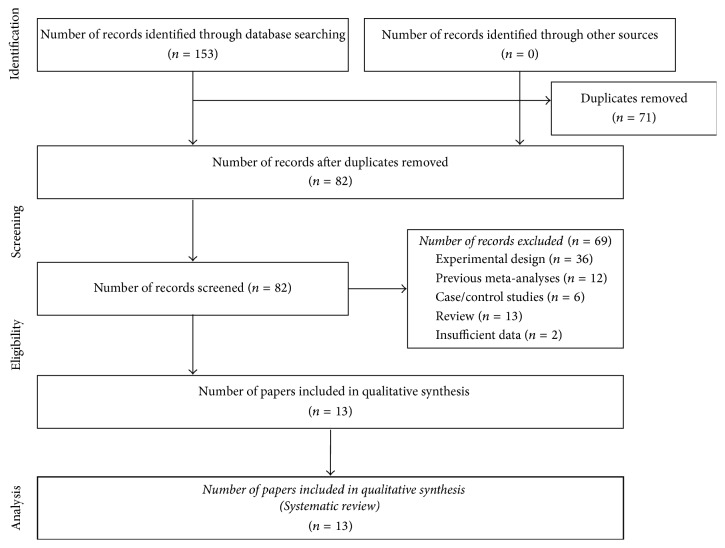
Flow diagram for inclusion of studies in systematic review.

*Search Strategy*. A systematic search in the Cochrane Library, Google Scholar, and Pubmed databases was performed for collection of studies previously published on October 2, 2016. A combination of the specific descriptors was used: “interleukin, or cytokine, or inflammatory Mediator”, “periodontitis, or periodontal disease, or chronic periodontitis, or aggressive periodontitis”, “polymorphism, or genetic variation”, and “meta-analysis”, as seen in Figure 1.

Three independent investigators reviewed all abstracts of the papers and their references to identify possible relevant studies. There was no restriction of language in the collection of articles. When the search resulted in studies of different authors which were published in distinct years but which addressed the same polymorphism the most recent study was selected for inclusion analysis and subsequent data extraction.

*Inclusion Criteria*. In order to be included in the research, the studies should show a clear association between polymorphisms in the inflammatory mediator gene and the risk in the development of periodontitis through meta-analyses. Following this, standard methods with allocation of patients in a case and control group data presented were dichotomous, using the fixed-effect or random-effect statistical models to calculate the Odds Ratio (OR) with values of *P* < 0.05 considered significant. Studies outside these criteria were excluded.

*Data Extraction*. Two calibrated investigators performed the data collection and discussion following a standardized form by first author, year of publication, polymorphism analyzed, number of cases and controls, OR value, and type of periodontitis analyzed.

## 3. Results and Discussion

At the end of the systematic search (Figure 1) 13 meta-analysis articles on 25 polymorphisms in seven interleukins (IL-1A, IL-1B, IL-4, IL-6, IL-8, IL-10, and IL-18), three cellular polymorphisms (Fc*γ* receptors: FCGR2A, FCGR3A, and FCGR3B), and five inflammatory mediators (COX-2, MMP-2, MMP-3, MMP-8, MMP-8, and MMP-9) comprised 71.531 participants (32.011 patients and 39.520 controls), as shown in Table 1. The studies were published in the period from 2012 to 2016. In addition, the results on heterogeneity as well as details about statistical characteristics of studies included in the systematic review are shown in Table 2.

In the pathophysiology of periodontitis, several inflammatory mediators contribute to damage of periodontal sites, destruction of supporting connective tissue, and loss of alveolar bone. An example of such mediators is interleukin 1, a soluble molecule involved in the host's immune response against microbial agents by signalling to neutrophilic infiltrate and tissue destruction due to increased secretion of matrix metalloproteinases [10].

Five meta-analysis studies were found for polymorphisms in two IL-1 variants (IL-1A and IL-1B) [15, 18–21]. The meta-analysis performed by Silva et al. [15] demonstrated that the −889 C/T polymorphism was significantly associated with a higher risk of chronic periodontitis in the general analysis (Table 1); however, the authors did not find a significant association for this polymorphism in the Brazilian population (OR = 0.97, 95% CI: 0.70, 1.35, and *P* = 0.87).

Such contradictory results can be explained by the greater heterogeneity in the genetic pattern of the Brazilian population [27]. Wang et al. [18], on the other hand, performed a meta-analysis with studies on this same polymorphism but focused on aggressive periodontitis, finding a nonsignificant association of this genetic variant with the disease in its aggressive form (*P* = 0.98). Aggressive and chronic periodontitis have distinct pathophysiological mechanisms; chronic and aggressive periodontitis can not be differentiated as to their histopathological changes [28] or type of microbial colonization [29]. However there is evidence that there are molecular and immunological differences between them, the presence of neutrophil abnormalities [30] and an increase in B lymphocytes in aggressive form being observed [31]; so approaches on these disease classifications are required [16].

The polymorphisms −511 C/T and +3954 C/T in the IL-1B gene were not significantly associated with the risk of developing either chronic [19] or aggressive periodontitis [20]. In contrast, the +3954 C/T polymorphism was associated with a higher risk of developing chronic periodontitis in a meta-analysis based in Asian population [21]. This meta-analysis composed of 20 case-control studies corroborates with data previously published in the literature [32]. One of the evidences that explain the association of polymorphisms in IL-1B only with chronic periodontitis is the fact that aggressive periodontitis is more like a genetically inherited disease and the IL-1 gene is not related to the specify genes [20]. The IL-1B can promote the movement of inflammatory cells from the blood to inflamed tissues, regulate the extracellular matrix, and induce other cytokines and IL-1B gene polymorphisms were previously reported to be associated with the severity of chronic periodontitis [21].

In IL-4, a proinflammatory cytokine is capable of inducing apoptosis of osteoblasts thus contributing to the progression of alveolar bone loss [9] and induces apoptosis in monocytes [17]; three polymorphisms (IL-4 −590 C/T (rs2243250), −33 C/T (rs2070874), and 70-bp) were addressed in a meta-analysis [17], which found, in most studies, no significant association between these genetic variants and the risk of developing periodontitis (Table 1). One of the possible explanations for such results is the potential publication that may have affected the evaluations for the 33-C/T and 70-pb polymorphisms because of the small number of studies for these polymorphisms (seven and four, resp.). In some studies the association between periodontitis and polymorphisms in IL-4 was observed in German, Brazilian, and Chinese patients. This can be explained by the association of polymorphisms in IL-4 with lack of monocyte downregulation, thus being a contributory factor to tissue breakdown in periodontitis [17].

Interleukin-6 (IL-6) is a potent mediator of bone resorption capable of stimulating macrophages and osteoclasts with increased tissue damage in both types of periodontitis evaluated [16]. The meta-analysis that evaluated the polymorphisms −174 G/C and −572 G/C in IL-6 showed that the two polymorphisms were associated with periodontitis. However, the results for the −174 G/C polymorphism had no association with the disease when analyzed for each ethnic group, except Brazilian population, and chronic periodontitis. The stratification by ethnicity and disease type indicated an association between the IL-6 −572 G allele and chronic periodontitis and periodontitis in Europeans [22]. To be associated with an increased inflammatory response, specifically in the presence of periodontopathogenic bacteria, the IL-6 gene may play a key role in the pathogenesis of periodontitis [22].

In another meta-analysis study addressing two polymorphisms in the IL-8 gene (Table 1), it was observed that the −251 A/T polymorphism was associated with the risk of developing periodontitis while the −845 C/T polymorphism was not significantly associated (*P* < 0.001, *P* = 0.40, resp.), because between-study heterogeneity was not evident. This meta-analysis evidenced that the IL-8 −251A/T polymorphism is related with a decreased risk of periodontitis in a Brazilian mixed population and an increased risk of periodontitis in an Asian population. The IL-8 gene has q13-q12 localization on chromosome 4, where the −251 A/T variant is related to an increase in the transcriptional activity of this cytokine [23]. The evidence that IL-8 −251A/T A allele is associated with higher expression of IL-8 can be observed in patients with chronic periodontitis in which higher IL-8 levels in the gingival crevicular fluid than healthy individuals were found. In addition, the expression of IL-8 in gingival epithelial cells can be induced by* Porphyromonas gingivalis* and* Tannerella forsythia* [23], bacteria commonly associated with periodontitis. It is important to note that although a very low genetic diversity was observed for the −845T/C polymorphism, the authors verified that Brazilian individuals with variant genotypes presented a higher risk of chronic periodontitis [23].

IL-10 is a suppressor cytokine that regulates negatively the immunological response of monocytes and macrophages. The meta-analysis [24] that evaluated polymorphisms in the IL-10 genes indicated that only the −592 C/A polymorphism in IL-10 is significantly associated with the high risk of disease development in the overall population (Table 1). However, in the same meta-analysis the IL-10 −819 gene polymorphism demonstrated association with chronic periodontitis onset in Caucasian population, suggesting a possible role of ethnic differences in genetic backgrounds besides showing that the IL-10 −1082 gene polymorphism did not indicate an association with either chronic periodontitis or aggressive periodontitis [24].

Data from the meta-analysis on polymorphisms in this interleukin corroborates with previously published data where there was also no association of these genetic variations with periodontitis, in both forms [33]. It has been proposed that IL-10 may attenuate periodontal tissue destruction through the induction of tissue inhibitors of metalloproteinases and the inhibitor of osteoclastogenesis [34].

Although there was no significant association in the evaluation calculations of the polymorphisms in the IL-18 gene and periodontitis, the meta-analysis developed by Li et al. [25] found a significant association of the polymorphisms (−607A>C and −137G>C) with high levels of this cytokine in the plasma of the patients with the disease when compared to the controls. The mechanisms by which IL-18 acts in periodontitis remain unknown, but they are indicating that polymorphisms in the IL-18 gene may alter the levels of this interleukin in the blood and thus contribute to the progression of the disease [25].

The findings suggest that the FCGR2A H131R allele and the FCGR3A F158V polymorphisms may be associated in the development of periodontitis. However, the associations between FCGR2A, FCGR3A, and FCGR3B polymorphisms and chronic periodontitis are susceptible due to a causal association or imbalance with the true polymorphism causing the disease not yet determined [10]. Already, the FCGR3B NA1/NA2 polymorphism has shown being associated with aggressive periodontitis. However, no relationship was identified between FCGR3A F158V and periodontitis [10]. The microorganisms antigens that have been opsonized with antibody can be either phagocytized via FcyRs on neutrophils or internalized via FcyRs by antigen-presenting cells (dendritic cells, monocytes, macrophages, and B cells). Therefore, any alteration in FcyR expression and function would alter host immune responses against periodontal pathogens and hence susceptibility to periodontal diseases [35].

Evaluating polymorphisms in COX-2 and MMP-2, MMP-3, MMP-8, and MMP-9 genes, two meta-analysis are available in literature [8, 11]. There are no significant associations between polymorphisms in COX-2 gene and periodontitis, as showed in Table 1. Nevertheless, contradicting findings are brought by other authors [8] who found an association between COX-2 and periodontitis in women of northern Indian population [36] and in the population of Northwestern European ethnicity [37]. The substitution of C/G at position −765 and substitution of A/G at position −1195, as well as substitution of C/T at position 8473 in the COX-2, were assumed to be polymorphisms associated with periodontitis [38].

As showed in Table 1 the −753 C/T polymorphism in MMP-2 and the −799 C/T polymorphism in MMP-8 are associated with both forms of periodontitis evaluated (aggressive and chronic forms). The −1171 A5/A6 polymorphism and −1562 C/T polymorphism in MMP-3 and MMP-9, respectively, are associated only with chronic form. This finding contradicts results about these polymorphisms in MMP-3 and MMP-9 in Chinese population with chronic periodontitis [39]. With these MMPs and others such as MMP-13 being considered as important inflammatory mediators with key role during periodontitis tissue damage [40] more studies are required to highlight the influence of MMP's polymorphisms in periodontitis.

## 4. Conclusion

In conclusion, this systematic review consisted of 13 meta-analysis articles with 25 polymorphisms in seven interleukins (IL-1A, IL-1B, IL-4, IL-6, IL-8, IL-10, and IL-18), three cellular polymorphisms (Fc*γ* receptors: FCGR2A, FCGR3A, and FCGR3B), and five inflammatory mediators (COX-2, MMP-2, MMP-3, MMP-8, MMP-8, and MMP-9) which comprised 71.531 participants (32.011 patients and 39.520 controls), approaching different classifications of the disease and demonstrating that polymorphisms in the IL-1A, IL-1B, IL-6, IL-10, MMP-3 (chronic form), and MMP-9 (chronic form) genes were significantly associated with risk of development of the disease, while other polymorphisms in the IL-4, 18, Fc*γ*, COX-2, MMP-2, MMP-3 (aggressive form), MMP-8, and MMP-9 (aggressive form) receptors were not significantly associated with periodontitis development.

## Figures and Tables

**Table 1:** Main characteristics and results of the studies in meta-analysis published in the period from 2012 to 2016 and included in systematic search.

Med	Author and reference	Year	*N*	Polymorphisms	Cases/controls	OR (IC 95%, *P*_value_)	Periodontitis classification
IL-1A	Wang et al. [18]	2014	19	−889 C/T	1,266/2,134	1.00 (0.88–1.14, *P* = 0.98)	Aggressive
	Silva et al. [15]	2017	23	−889 C/T	2,174/1,756	1.22 (1.09–1.36, *P* < 0.0004)	Chronic

IL-1B	Zeng et al. [19]	2015	19	−511 C/T	2,173/3,900	1.03 (0.72–1.46, *P* = 0.88)	Chronic
	Chen et al. [20]	2015	25	+3954 C/T	1,594/2,483	0.99 (0.79–1.23, *P* = 0.91)	Aggressive
	Ma et al. [21]	2015	20	+3954 C/T	1,656/1,498	1.60 (1.06–2.42, *P* = 0.02)	Chronic

IL-4	Yan et al. [17]	2015	12 7 4	−590 C/T −33 C/T 70 pb	1,220/2,039 715/967 426/506	1.20 (0.78–1.85, *P* = 0.42) 0.97 (0.60–1.58, *P* = 0.91) 1.26 (0.76–2.11, *P* = 0.37)	Aggressive Chronic

IL-6	Song et al. [22]	2013	16 10	−174 G/C −572 G/C	1,887/1,490 1,436/998	1.18 (0.91–1.55, *P* = 0.20) 1.52 (1.15–2.00, *P* = 0.003)	Aggressive Chronic

IL-8	Chen et al. [23]	2015	11 3	−251 A/T −845 T/C	1,555/2,130 678/525	0.89 (0.70–1.12, *P* < 0.001) 1.41 (0.99–2.01, *P* = 0.40)	Aggressive Chronic

IL-10	Zhong et al. [24]	2012	5 7 6	−1082 A/G −819 C/T −592 C/A	1,105/976 749/603 673/587	0.96 (0.73–1.26, *P* = 0.77) 1.13 (0.96–1.33, *P* = 0.16) 1.38 (1.04–1.85, *P* = 0.03)	Aggressive Chronic

IL-18	Li et al. [25]	2014	3 3	−607 A/C −137 G/C	576/458 576/458	1.86 (1.30–2.65, *P* = 0.192) 1.66 (1.37–2.02, *P* = 0.281)	Aggressive Chronic

FCGR2A FCGR3A FCGR3B	Song and Lee [10]	2013	7 6 1 3 3 3	R131H F158V NA1/NA2	1,626/1,496 7,68/1,256 178/128 434 512668/490 320/364	0.947 (0.814–1.103, *P* = 0.485) 1.112 (0.918–1.348, *P* = 0.278) 1.371 (0.810–2.320, *P* = 0.240) 0.940 (0.673–1.315, *P* = 0.719) 1.026 (0.806–1.305, *P* = 0.836) 2.138 (0.956–4.784, *P* = 0.064)	Chronic Aggressive Chronic Aggressive Chronic Aggressive

COX-2	Jiang et al. [8]	2014	5 2 3 1 2	−765G/C −1195G/A 8473 T/C	1,913/2,984 604/1192 1,231/2,138 519/1036 202/208	1.11 (0.79–1.55, *P* > 0.005) 1.15 (0.81–1.64, *P *> 0.005) 0.40 (0.03–5.44, *P *> 0.005) 1.25 (0.87–1.80, *P *> 0.005) 0.65 (0.46–0.91, *P *> 0.005)	Chronic Aggressive Chronic Aggressive Chronic

MMP-2 MMP-3 MMP-8 MMP-9	Weng et al. [11]	2016	2 2 6 1 2 2 7 2	−753 C/T −1171 A5/A6 −799C/T −1562 C/T	236/234 171/280 697/1,530 37/142 702/384 1,96/167 859/1,186 191/285	1.28 (1.07–1.54 *P *> 0.005) 2.01 (0.98–4.11, *P *> 0.005) 1.44 (1.25–1.66, *P *< 0.001) 1.54 (0.82–2.89, *P* = 0.175) 1.28 (1.07–1.54, *P *> 0.005) 2.01 (0.98–4.11, *P *> 0.005) 0.49 (0.31–0.77, *P* = 0.002) 0.93 (0.67–1.29, *P* = 0.656)	Chronic Aggressive Chronic Aggressive Chronic Aggressive Chronic Aggressive

Med: inflammatory mediator, IL: interleukins, FGCGR: receptor Fc*γ*, COX-2: cyclooxygenase 2, MMP: matrix metalloproteinase, *N*: number of studies included in meta-analysis available, OR: Odds Ratio for the allelic evaluation (mutants allelic × wild allelic comparison), IC: confidence interval, pb: bases pair, and *P* < 0.05: statistic value significant.

**Table 2:** Statistical characteristics and results about heterogeneity value of the studies in meta-analysis and included in systematic search.

Med	Author and reference	Polymorphism	*I* ^2^ (%)	*P*_heterogeneity_	Statistical model used	Periodontitis classification
IL-1A	Wang et al. [18]	−889 C/T	28.86	*P* > 0.05	FE	Aggressive
	Silva et al. [15]	−889 C/T	15	*P* = 0.28	FE	Chronic

IL-1B	Zeng et al. [19]	−511 C/T	79.54	*P* < 0.05	RE	Chronic
	Chen et al. [20]	+3954 C/T	62.2	*P* < 0.05	RE	Aggressive
	Ma et al. [21]	+3954 C/T	81%	*P* < 0.05	RE	Chronic

IL-4	Yan et al. [17]	−590 C/T −33 C/T 70 pb	90.4 85.7 75.1	*P* < 0.05 *P* < 0.05 *P* < 0.05	RE RE RE	Aggressive/chronic

IL-6	Song et al. [22]	−174 G/C −572 G/C	64.9 61.8	*P* < 0.05 *P* < 0.05	RE RE	Aggressive/chronic

IL-8	Chen et al. [23]	−251 A/T −845 T/C	NR NR	*P* < 0.001 *P* = 0.40	RE FE	Aggressive/chronic

IL-10	Zhong et al. [24]	−1082 A/G −819 C/T −592 C/A	23.9 29.8 56.6	*P* = 0.26 *P* = 0.20 *P* = 0.04	FE FE RE	Aggressive/chronic

IL-18	Li et al. [25]	−607 A/C −137 G/C	38.1 0	*P* = 0.19 *P* = 0.97	FE FE	Aggressive/chronic

FCGR2A FCGR3A FCGR3B	Song and Lee [10]	R131H F158V NA1/NA2	5.95 0 NA 0 0 84.7	*P* = 0.45 *P* = 0.49 NA *P* = 0.48 *P* = 0.56 *P* = 0.001	FE FE NA FE FE RE	Chronic Aggressive Chronic Aggressive Chronic Aggressive

COX-2	Jiang et al. [8]	−765G/C −1195G/A 8473 T/C	95.4 96.7 75.9 NA 0	*P* < 0.05 *P* < 0.05 *P* < 0.05 NA *P* > 0.05	RE RE RE NA FE	Chronic Aggressive Chronic Aggressive Chronic

MMP-2 MMP-3 MMP-8 MMP-9	Weng et al. [11]	−753 C/T −1171 A5/A6 −799C/T −1562 C/T	0 29.9 34.4 NA 0 85.3 85.8 0	*P* > 0.05 *P* > 0.05 *P* = 0.17 NA *P* > 0.05 *P* < 0.05 *P* < 0.001 *P* = 0.49	FE FE FE NA FE RE RE FE	Chronic Aggressive Chronic Aggressive Chronic Aggressive Chronic Aggressive

Med: inflammatory mediator, IL: interleukins, FGCGR: receptor Fc*γ*, COX-2: cyclooxygenase 2, MMP: matrix metalloproteinase, *I*^2^: chi-squared based *Q* statistic test, NR: not reported, NA: not applicable, and *P* < 0.05: statistic value significant.
